# Higher serum tissue inhibitor of metalloproteinase-1 predicts atrial fibrillation recurrence after radiofrequency catheter ablation

**DOI:** 10.3389/fcvm.2022.961914

**Published:** 2022-10-13

**Authors:** Haiwei Li, Weiping Sun, Zefeng Wang, Ziyu Wang, Xiao Du, Junjun Chen, Jianwei Gao, Xuxia Liu, Xipeng Wang, Yueli Wang, Yongquan Wu, Xiaoping Zhang

**Affiliations:** ^1^Beijing Anzhen Hospital, Capital Medical University, Beijing, China; ^2^Beijing Institute of Heart, Lung and Blood Vessel Disease, Beijing, China; ^3^The Key Laboratory of Remodeling-Related Cardiovascular Diseases, Ministry of Education, Beijing, China; ^4^Beijing Children’s Hospital, Capital Medical University, National Center for Children’s Health, Beijing, China; ^5^Department of Cardiology, Beijing Anzhen Hospital, Capital Medical University, Beijing, China

**Keywords:** atrial fibrillation recurrence, tissue inhibitor of metalloproteinase-1, radiofrequency catheter ablation, atrial fibrillation, extracellular matrix

## Abstract

**Background:**

Tissue inhibitor of metalloproteinase-1 (TIMP-1) levels is strongly associated with cardiac extracellular matrix accumulation and atrial fibrosis. Whether serum levels of TIMP-1 are associated with atrial fibrillation (AF) recurrence following radiofrequency catheter ablation (RFCA) remains unknown.

**Materials and methods:**

Serum TIMP-1 levels of patients with AF before they underwent initial RFCA were measured using ELISA. Univariate and multivariate-adjusted Cox models were constructed to determine the relationship between TIMP-1 levels and AF recurrence. Multivariate logistic regression analyses were performed to determine predictors of AF recurrence.

**Results:**

Of the 194 enrolled patients, 61 (31.4%) had AF recurrence within the median 30.0 months (interquartile range: 16.5–33.7 months) of follow-up. These patients had significantly higher baseline TIMP-1 levels than those without AF recurrence (129.8 ± 65.7 vs. 112.0 ± 51.0 ng/ml, *P* = 0.041). The same was true of high-sensitivity C-reactive protein (3.9 ± 6.0 vs. 1.9 ± 2.8 ng/ml, *P* = 0.001). When a TIMP-1 cutoff of 124.15 ng/ml was set, patients with TIMP-1 ≥ 124.15 ng/ml had a higher risk of recurrent AF than those with TIMP-1 < 124.15 ng/ml (HR, 1.961, 95% CI, 1.182–2. 253, *P* = 0.009). Multivariate Cox regression analysis revealed that high TIMP-1 was an independent risk factor for AF recurrence. Univariate Cox regression analysis found that substrate modification surgery does not affect AF recurrence (*P* = 0.553). Subgroup analysis revealed that female sex, age < 65 years, hypertension (HTN), body mass index (BMI) ≥ 24 kg/m^2^, CHA2DS2-VASc score < 2, HAS-BLED score < 3, and EHRA score = 3 combined with high TIMP-1 level would perform well at predicting AF recurrence after RFCA.

**Conclusion:**

Elevated preoperative TIMP-1 levels are related to a higher risk of AF recurrence and can independently predict AF recurrence following RFCA.

## Introduction

Atrial fibrillation (AF) is a common cardiac arrhythmia (1). Patients with AF face a high risk of stroke, left ventricular (LV) diastolic dysfunction, heart failure, and sudden cardiac death, which seriously compromise their quality of life. The elderly population is most susceptible to AF, which has increased their rate of mortality, morbidity, and disability (2).

Radiofrequency catheter ablation (RFCA) has become the first-line therapy in patients with symptomatic, drug-resistant AF (3). AF recurrence is a major complication after RFCA, and the rates of AF recurrence are estimated to range from 24 to 45% following ablation (4, 5). AF recurrence after RFCA is defined as AF, atrial flutter, or atrial tachycardia for 30 s or more after a 3-month blanking period without antiarrhythmic drugs (3, 5).

Evidence suggests that atrial remodeling and atrial fibrosis are correlated with AF progression (2, 6, 7). Atrial fibrosis is the process by which extracellular matrix (ECM) is deposited within the atria, and ECM turnover is regulated by the balance of matrix metalloproteinases (MMPs) and tissue inhibitors of metalloproteinases (TIMPs) (8, 9). Some specific molecular markers of fibrosis increase with higher arrhythmia burden (10, 11), such as tissue inhibitor of metalloproteinase-1 (TIMP-1) (12), matrix metalloproteinase 9 (MMP-9) (13), and bone morphogenic protein-10 (BMP10) (14). Previous studies showed that high plasma concentrations of TGF-β and TIMP-1 and low ejection fraction were closely related to the voltage and volume of the left atrium (LA) in patients with non-valvular AF (15). Some data suggest that plasma TIMP-1 concentrations less than 107 ng/ml combined with the absence of a variant allele at rs10033464 might predict a lower rate of paroxysmal AF recurrence after RFCA (16).

Despite the importance of atrial fibrosis being associated with a higher risk of recurrent AF in patients (9), less is known about whether some biomarkers for fibrosis provide additional predictive value in risk stratification for disease severity and AF recurrence. Therefore, we hypothesized that the serum concentration of TIMP-1 can be used to predict AF recurrence in patients. The purpose of this study was to define the association of serum TIMP-1 concentration with AF recurrence in patients after RFCA.

## Materials and methods

### Study design and population

**FIGURE 1:**
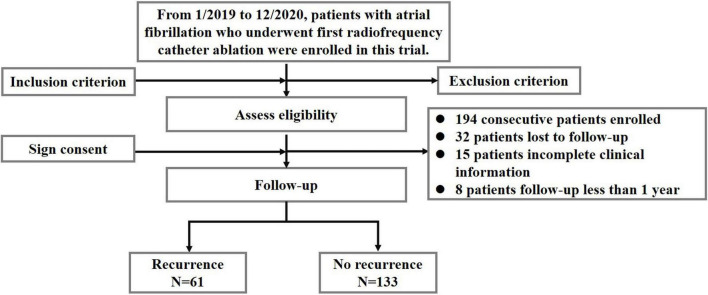
Flow diagram showing population selection and study design. The algorithm of patients included in this study after excluding patients not meeting the inclusion criteria. Refer to the text for details with regard to the exclusion criteria.

This study is a prospective cohort study, which comprised 249 patients with AF who underwent their initial RFCA in the Department of Cardiology of Beijing Anzhen Hospital between January 2019 and December 2020. The flowchart of this study is shown in Figure 1.

The inclusion criteria were as follows: (1) enrolling patients > 18 years of age; (2) enrolling patients who were diagnosed AF and underwent RFCA treatment for the first time; and (3) voluntary participation in this study and signed informed consent.

The exclusion criteria were as follows: (1) history or findings of cardiovascular disease, including heart failure symptoms, abnormal cardiac structural disease, and history of coronary artery bypass graft surgery (CABG), cryoballoon ablation, and other cardiac surgeries; (2) other diseases including mental disease, severe renal dysfunction, advanced malignant tumor, acute and chronic inflammatory diseases, and autoimmune diseases; (3) pregnancy; and (4) LA anteroposterior diameter > 50 mm.

This study was designed and performed in accordance with the Declaration of Helsinki for Human Research and was approved by the Beijing Anzhen Hospital Ethics Committee (Approval No: 2022042X). Informed consent was obtained from all participants.

### Data collection

The following demographic and clinical data of all patients were collected: (1) general clinical data: age, gender, body mass index (BMI, kg/m^2^), type of AF, comorbidities and calculation of EHRA score, CHA2DS2-VASc score, HAS-BLED score, and history of medication and echocardiographic parameters (preoperative measurements were performed with a Philips 7C color Doppler ultrasound); (2) hematological indices (results of fasting blood sample obtained on the latest preoperative morning): white blood cell (WBC), red blood cell (RBC), platelet count (PLT), hemoglobin (Hb), creatinine (CREA), fasting blood glucose (Glu), glycated albumin (GA), homocysteine (HCY), alanine aminotransferase (ALT), aspartate transaminase (AST), gamma-glutamyl transpeptidase (GGT), total protein (TP), albumin (Alb), globulin (Glo), total bilirubin (Tbil), triacylglycerol (TG), total cholesterol (Tcho), and low-density lipoprotein cholesterol (LDL-c). Three biomarkers were measured including high-sensitivity C-reactive protein (hs-CRP), B-type natriuretic peptide (BNP), and D-dimer.

### Blood sampling and enzyme-linked immunosorbent assay

Serum samples separated from peripheral venous blood were obtained and processed on the day prior to RFCA. Serum TIMP-1 levels were determined using an enzyme-linked immunosorbent assay (ELISA) (Bioss, # bsk11100, Beijing, China). The 96-well microtiter plates were coated with diluted capture TIMP-1 primary antibody and incubated overnight at 4°C. After being washed and blocked, the plates were ready to add diluted samples or standards and incubated for 2 h at room temperature (RT). Then, the detection antibody was incubated for 1 h, and streptavidin-HPR was incubated for 20 min at RT. The 3,3’,5,5’-tetramethylbenzidine (TMB) liquid substrate solution was added and incubated in the dark for 15 min. The color reaction was arrested by adding a stop solution. Optical density was immediately measured using a microplate reader at a wavelength of 450 nm. A standard curve was constructed by plotting absorbance for standard samples, and then the TIMP-1 concentration of the serum sample was calculated.

### Radiofrequency catheter ablation

All patients underwent transesophageal echocardiography to exclude atrial thrombus before RFCA. After written informed consent was obtained from patients, ablation procedures were performed under local anesthesia with mild conscious sedation. Circumferential pulmonary vein isolation (CPVI) was the initial part of ablation in all participants and was achieved by circumferential ablation around PV ostia. Intravenous heparin was administered continuously to maintain an activated clotting time between 300 and 350 s after the transseptal puncture. A mapping catheter (PentaRay^®^; Biosense Webster, Diamond Bar, California, United States) and an ST ablation catheter (Thermocool smarttouc^*h*®^; Biosense Webster, Diamond Bar, California, United States) were inserted into the LA through non-steerable long sheathes, followed by 3-dimensional mapping conducted using PentaRay. CPVI was performed using irrigated ablation catheters (Thermocool Smarttouch; Biosense Webster, Diamond Bar, California, United States) in a power control mode at 35 W (irrigation flow 17 ml/min). The procedural endpoint of ablation was AF termination. Selective additional atrial ablation (i.e., cavotricuspid isthmus ablation, superior vena cava isolation, or LA linear ablation) was only performed in patients with AF persisting despite completion of CPVI according to the mapping results [selective ablation of complex-fractionated atrial electrograms, atrial low-voltage sites, or other atrial arrhythmias (atrial flutter and atrial tachycardia)]. The electrophysiological endpoint of CPVI was a bidirectional conduction block between the LA and PVs.

### Follow-up

Regular follow-up visits in outpatient clinics were conducted at 3, 6, 12, 24, and 36 months. At each visit, a detailed medical and physical examination, 12-lead electrocardiogram (ECG), and 24-h Holter monitoring were performed. They were strongly recommended to visit the nearest hospital for an ECG if they felt symptoms that could be attributed to arrhythmia or noticed any irregularity of their peripheral pulse by routine self-measurement. The outcome was AF recurrence defined as any documented atrial tachyarrhythmia (AF, atrial flutter, or atrial tachycardia) episode lasting for at least 30 s after ablation, excluding a 3-month blanking period.

Follow-up records were based on telephone interviews, and patients’ regular visits to outpatient clinics at the Beijing Anzhen Hospital and outcomes were adjudicated by trained study personnel and cardiologists.

### Statistical analysis

Continuous variables with normal distribution are expressed as mean ± standard deviation (SD), and non-normally distributed variables were described as the median and interquartile range (IQR). Comparisons of means between groups were analyzed using the independent sample *t*-test for normally distributed data and the Mann-Whitney *U*-test for non-normally distributed data. Categorical data were presented as frequencies or percentages and compared between groups using the chi-squared test or Fisher’s exact test, as appropriate. Univariate and multivariate Cox regression analyses were performed to determine risk factors for AF recurrence, and the hazard ratio (HR) and 95% CI were calculated. Variables with values of *P* < 0.05 in the univariate analysis were included in the multivariate analysis. Time-dependent survival between groups (TIMP1 and hs-CRP) was evaluated using Kaplan-Meier curves and the log-rank test. To enhance the ability of biomarkers predicting AF recurrence after ablation, a receiver operating characteristic (ROC) curve was constructed, and the area under the curve (AUC) best cutoff value was calculated. Stratified analyses were also performed using the following variables: age (≥ 65 vs. < 65 years), BMI (≥ 24 kg/m^2^ vs. < 24 kg/m^2^), sex, hypertension (HTN), diabetes, coronary artery disease, LV ejection fraction (≥ 50 vs. < 50%), hypertriglyceridemia (> 1.7 mmol/L), and hyperhomocysteinemia (> 15 μmol/L). Multiplicative interactions were calculated in each subgroup. All data were analyzed using the SPSS 20.0 (IBM Corp., Armonk, NY, USA), R (version 4.0.4), and GraphPad Prism 6.0 software (CA, USA). Two-tailed *P*-values of < 0.05 were considered statistically significant.

## Results

### Patient clinical characteristics

From January 2019 to December 2020, we enrolled 249 patients with AF who underwent their initial RFCA at the Department of Cardiology of Beijing Anzhen Hospital in the study. We excluded 55 patients, i.e., 32 patients were lost to follow-up, 15 patients had incomplete clinical information, and 8 patients had a follow-up of less than 1 year (Figure 1).

All patients were followed up for at least 12 months following ablation. Of the 194 patients with AF (aged 59.9 ± 10.2 years, 66.5% male) included in our analyses, 61 (31.4%) experienced AF recurrence after ablation, as shown in Figure 1.

The baseline clinical characteristics of the participants are shown in Tables 1, 2. As shown in Table 1, more men underwent AF ablation than women (66.5% vs. 33.5%), but the proportion of women in the recurrence group was higher than that in the non-recurrence group (44.3 vs. 28.6%, *P* < 0.05). A total of 94 people underwent substrate modification surgery (recurrence group 31 (50.8%) vs. no-recurrence group 63 (47.4%), *P* = 0.657). We found no significant differences in clinical characteristics, including age, BMI, medical history, AF-related score, echocardiographic parameters, and laboratory examination (Table 2) between the recurrence and non-recurrence groups.

**TABLE 1:** Baseline clinical characteristics of the participants stratified according to post-ablation AF recurrence.

Characteristic	Total (194)	Recurrence (61)	No recurrence (133)	*P* value
**Demographics**				
Age, years	59.9 ± 10.2	59.5 ± 9.6	60.0 ± 10.4	0.76
Gender (M, %)	129(66.5)	34(55.7)	95(71.4)	0.032
BMI, kg/m^2^	26.6 ± 4.2	26.6 ± 4.1	26.6 ± 4.2	0.993
Duration of AF, months	40.6 ± 44.4	44.1 ± 45.1	38.9 ± 44.2	0.447
**Type of atrial fibrillation**				0.19
Paroxysmal, *n*(%)	93(47.9)	25(41.0)	68(51.1)	
Persistent, *n*(%)	101(52.1)	36(59.0)	65(48.9)	
**Medical history**				
CAD, %	21(10.8)	6(9.8)	15(11.3)	0.765
HTN, %	102(52.6)	31(50.8)	71(53.4)	0.741
DM, %	27(13.9)	7(14.5)	20(15.0)	0.507
Stroke, %	15(7.7)	6(9.8)	9(6.8)	0.459
Smoking, %	54(27.8)	12(19.7)	42(31.6)	0.087
Drinking, %	12(6.2)	3(4.9)	9(6.8)	0.622
**Drug administration**				
Diuretic, %	4(2.1)	0	4(3.0)	0.172
Statins, %	43(22.2)	12(19.7)	31(23.3)	0.572
ACEI/ARB, %	39(20.1)	15(24.6)	24(18)	0.292
CCB, %	25(12.9)	8(13.1)	17(12.8)	0.949
Beta-block, %	31(16.0)	10(16.4)	21(15.8)	0.915
**AF related score**				
EHRA score				0.736
1, *n*(%)	15(7.7)	6(9.8)	9(14.8)	
2, *n*(%)	108(55.7)	33(54.1)	75(56.4)	
3, *n*(%)	71(36.5)	22(36.1)	49(36.8)	
CHA2DS2-VASc score				0.621
0 or 1, *n*(%)	110(56.7)	33(54.1)	77(57.9)	
≥2, *n*(%)	84(43.3)	28(45.9)	56(42.1)	
HAS-BLED score				0.425
≥3, *n*(%)	10(5.2)	2(3.3)	8(6.0)	
Substrate modification, *n*(%)	94(48.5)	31(50.8)	63(47.4)	0.657
**Echocardiography**				
LAd, mm	40.9 ± 5.6	40.5 ± 6.0	41.1 ± 5.4	0.478
LVEF, %	62.0 ± 5.5	62.3 ± 5.6	61.9 ± 5.4	0.665
LVEDD, mm	47.9 ± 4.5	47.1 ± 4.9	48.2 ± 4.2	0.118
LVESD, mm	31.5 ± 4.1	31.2 ± 4.7	31.7 ± 3.9	0.457

AF, atrial fibrillation; BMI, body mass index; CAD, coronary artery disease; HTN, hypertension; DM, diabetes mellitus; ACEI, angiotensin-converting enzyme inhibitors; ARB, angiotensin receptor blocker; CCB, calcium channel blocker; LAd, left atrium diameter; LVEF, left ventricular ejection fraction; LVEDD, left ventricular end-diastolic dimension; LVESD, left ventricular end-systolic dimension.

**TABLE 2:** Baseline laboratory examination of the participants stratified according to post-ablation atrial fibrillation recurrence.

Characteristic	Total (194)	Recurrence (61)	No recurrence (133)	*P* value
RBC, 10^9^/l	4.8 ± 0.5	4.8 ± 0.7	4.7 ± 0.5	0.555
WBC, 10^9^/l	6.7 ± 1.5	6.7 ± 1.6	6.7 ± 1.5	0.916
PLT, 10^9^/l	217.0 ± 52.7	225.7 ± 47.7	212.9 ± 54.6	0.117
Hb, g/l	149.3 ± 18.8	149.5 ± 16.7	149.3 ± 19.8	0.935
CREA, umol/l	74.4 ± 40.3	67.1 ± 15.0	77.7 ± 47.3	0.089
GLU, mmol/l	5.8 ± 1.3	5.8 ± 1.1	5.8 ± 1.3	0.749
GA, %	14.5 ± 2.5	14.3 ± 2.3	14.6 ± 2.6	0.426
HCY, umol/l	15.6 ± 8.4	15.1 ± 10.1	15.8 ± 7.5	0.626
ALT, U/l	25.3 ± 20.0	27.9 ± 26.2	24.2 ± 16.4	0.234
AST, U/l	24.4 ± 15.0	25.0 ± 15.0	24.1 ± 15.0	0.693
GGT, U/l	34.8 ± 28.0	35.9 ± 29.6	34.3 ± 27.3	0.717
TP, g/l	71.5 ± 5.4	71.4 ± 4.9	71.6 ± 5.6	0.764
Alb, g/l	44.6 ± 4.3	44.5 ± 4.1	44.7 ± 4.3	0.768
Glo, g/l	26.9 ± 4.8	26.9 ± 3.6	26.9 ± 5.2	0.938
Tbil, umol/l	14.8 ± 6.9	15.2 ± 7.4	14.7 ± 6.6	0.654
TG, mmol/l	1.7 ± 1.2	1.4 ± 0.7	1.8 ± 1.4	0.078
Tcho, mmol/l	4.4 ± 1.0	4.5 ± 1.1	4.4 ± 0.9	0.428
LDL-c, mmol/l	2.7 ± 1.0	2.8 ± 1.1	2.7 ± 0.9	0.544

AF, atrial fibrillation; WBC, white blood cell; RBC, red blood cell; PLT, platelet count; Hb, hemoglobin; CREA, creatinine; Glu, fasting blood glucose; GA, glycated albumin; HCY, homocysteine; ALT, alanine aminotransferase; AST, aspartate transaminase; GGT, gamma-glutamyl transpeptidase; TP, total protein; Alb, albumin; Glo, globulin; Tbil, total bilirubin; TG, triacylglycerol; Tcho, total cholesterol; LDL-c, low-density lipoprotein cholesterol.

### Baseline TIMP-1 and hs-cRP level is positively correlated with atrial fibrillation recurrence

We measured the levels of TIMP-1 and hs-CRP using serum samples obtained from patients before AF ablation. Patients were assigned to two groups according to the recurrence of AF after the 3-month blanking period.

**FIGURE 2:**
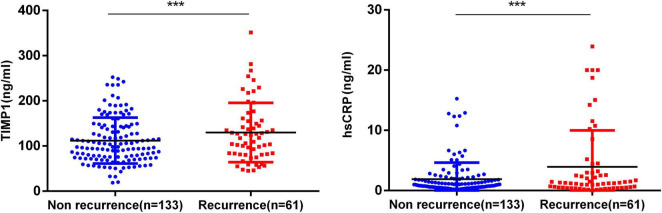
Circulating levels of tissue inhibitor of metalloproteinase-1 (TIMP-1) and high-sensitivity C-reactive protein (hs-CRP) in patients with atrial fibrillation (AF) recurrence compared with the non-recurrence group. ***P < 0.05.

Figure 2 compares TIMP-1 and hs-CRP concentrations between the AF recurrence and non-recurrence groups. The level of TIMP-1 in the recurrence group was significantly higher than that in the non-recurrence group (129.8 ± 65.7 ng/ml vs. 112.0 ± 51.0 ng/ml, *P* = 0.041), and participants with AF recurrence had a significantly higher level of hs-CRP than those without AF recurrence (3.9 ± 6.0 mg/L vs. 1.9 ± 2.8 mg/L, *P* < 0.05). Among the patients with AF, there was no statistically significant difference in the level of BNP (135.0 ± 123.7 vs. 121.4 ± 103.1, *P* = 0.449) or D-dimer (215.8 ± 794.1 vs. 111.0 ± 152.4, *P* = 0.145) between patients with AF recurrence and those without (Table 3).

**TABLE 3:** Baseline biomarkers of the participants stratified according to post-ablation atrial fibrillation recurrence.

Characteristic	Total (194)	Recurrence (61)	No recurrence (133)	*P* value
TIMP-1, ng/ml	117.6 ± 56.5	129.8 ± 65.7	112.0 ± 51.0	0.041
hsCRP, ng/ml	2.5 ± 4.2	3.9 ± 6.0	1.9 ± 2.8	0.001
BNP, pg/ml	125.6 ± 109.6	135.0 ± 123.7	121.4 ± 103.1	0.449
D-dimer, ng/ml	143.7 ± 461.6	215.8 ± 794.1	111.0 ± 152.4	0.145

AF, atrial fibrillation; TIMP-1, tissue inhibitors of metalloproteinase-1; hsCRP, high-sensitivity C-reactive protein; BNP, B-type natriuretic peptide.

### Clinical outcomes and subgroup analysis

**FIGURE 3:**
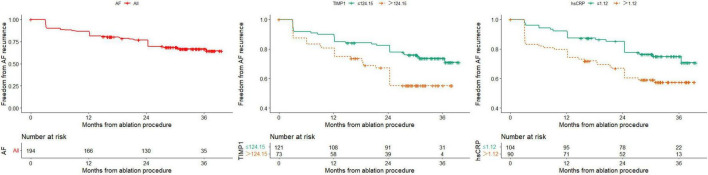
Kaplan-Meier survival curves for freedom from AF recurrence of all patients and stratified by TIMP-1 and hsCRP.

The median follow-up time after RFCA was 30.0 months (IQR: 16.5–33.7 months), and 61 patients experienced recurrence after AF ablation during the follow-up period. The Kaplan–Meier survival table showed that the rate of 12 months of freedom from AF recurrence was 85.4% (Figure 3), with paroxysmal AF at 90.3% and persistent AF at 80.8% (*P* = 0.053). The AUC of the continuous variables was calculated for the ROC, and the best cutoff value was selected as the boundary value of the categorical variables. Finally, the variables with *P* < 0.05 by univariate Cox analysis were TIMP-1, hs-CRP, PLT, and TCHO levels (Supplementary Table 1). We can also find from Supplementary Figure 1 whether undergoing substrate modification surgery does not affect AF recurrence.

Subsequently, we performed multivariate Cox analyses using the above variables, and the results showed that a high TIMP-1 level was an independent risk factor for AF recurrence (Table 4). The HR for AF recurrence was 1.793 (95% CI: 1.062–3.021, *P* = 0.029).

**TABLE 4:** Multivariate Cox analysis of risk factors for atrial fibrillation recurrence.

Parameter	Recurrence		
	HR (95% CI)	*P* value	Best cut-off value
Hs-CRP	1.513(0.883–2.593)	0.132	1.12
TIMP-1	1.793(1.062–3.021)	0.029	124.15
PLT	1.65(0.893–3.047)	0.11	196.5
TCHO	1.847(0.851–4.008)	0.121	5.94

AF, atrial fibrillation; TIMP-1, tissue inhibitors of metalloproteinase-1; hs-CRP, high-sensitivity C-reactive protein; PLT, platelet count; Tcho, total cholesterol.

The unadjusted models revealed the relationship between high TIMP-1 level and AF recurrence, and the HR of high TIMP-1 level in predicting AF recurrence was largely unchanged after model adjustment, as shown in Table 5.

**TABLE 5:** Adjusted hazard ratios of post-ablation AF recurrence by a high level of TIMP-1 (>124.15 ng/ml) relative to the level of TIMP-1 (≤124.15 ng/ml).

Model adjustment		Recurrence	
		HR (95% CI)	*P* value
Unadjusted	TIMP1	1.961(1.182–3.253)	0.009
Model 1	TIMP1	2.228(1.309–3.79)	0.003
Model 2	TIMP1	2.28(1.289–4.034)	0.005
Model 3	TIMP1	2.052(1.118–3.767)	0.02

AF, atrial fibrillation; TIMP-1, tissue inhibitors of metalloproteinase-1. Model 1: adjusted for age (≥65 years, <65 years), sex. Model 2: adjusted for age, sex, hypertension (HTN), diabetes, CAD, stroke, smoking, and drinking. Model 3: adjusted for age, sex, HTN, diabetes, CAD, stroke, smoking, drinking, LVEF (≥50%, <50%), BMI (≥24, <24 kg/m^2^), type of AF, AF duration, and substrate modification.

**FIGURE 4:**
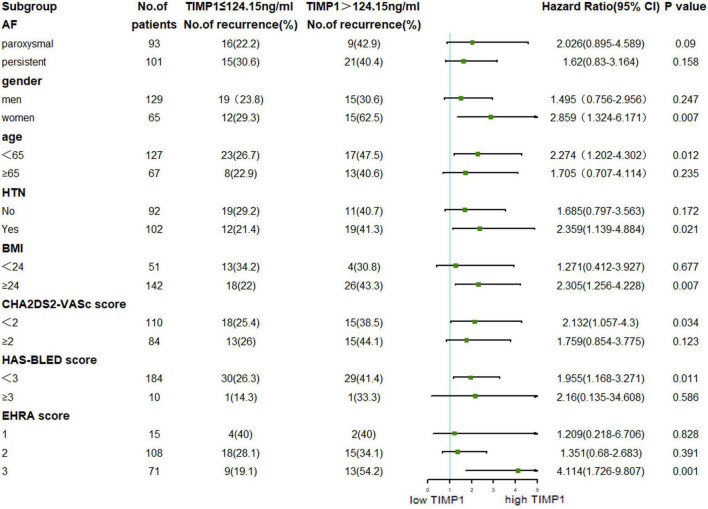
Based on serum TIMP-1 level for atrial fibrillation recurrence in different subgroups.

Subgroup analyses of participants based on serum TIMP-1 level and age, sex, type of AF, BMI, HTN, and AF-related score were carried out to determine the factors associated with AF recurrence. From Figure 4, we found that female sex, age < 65 years, HTN, BMI ≥ 24 kg/m^2^, CHA2DS2-VASc score < 2, HAS-BLED score < 3, and EHRA score = 3 combined with high TIMP-1 level could better predict AF recurrence after RFCA. No significant difference was found in other subgroup comparisons.

## Discussion

In this study, we prospectively explored the predictive value of the TIMP-1 level for AF recurrence after RFCA. We found that patients with AF who had elevated preoperative baseline levels of TIMP-1 had an increased rate of recurrence. These findings were consistently observed whether TIMP-1 levels were analyzed as continuous data or dichotomized around a cutoff value of 124.15 ng/ml, indicating that the present results are robust and do not depend on a certain cutoff point. In this study, further univariate Cox regression analysis revealed that high TIMP-1, hs-CRP, PLT, and TCHO levels were significantly associated with AF recurrence after RFCA. Multivariate Cox regression analysis showed that a high TIMP-1 level was an independent risk factor for AF recurrence.

Atrial fibrosis is a pathological process of AF structural remodeling (17). Atrial fibrosis in AF causes an imbalance in ECM formation and degradation. In profibrotic stimuli, the homeostatic balance in the ECM was disrupted by an increase in ECM formation over degradation. TIMPs are thought to regulate ECM remodeling through direct inhibition of MMP-dependent ECM proteolysis. Four TIMPs and 23 MMPs have been described in humans and play roles in cardiac fibrosis (18). A study evaluating atrial remodeling in aortic stenosis patients with chronic AF showed a decrease in the MMP16/TIMP4 ratio in patients with AF along with an increased serum TIMP1 and TIMP2 proteins (7). Prior studies have associated high plasma TGF-β1 and TIMP-1 levels with the electroanatomical remodeling of the LA in patients with non-valvular AF (15). Chromosome 4q25 variant alleles and TIMP-1 levels are also associated with clinical outcomes in those with paroxysmal AF (16). Our study showed that TIMP-1 is an independent risk factor for AF recurrence following RFCA. These studies have yielded meaningful results and may provide predictive value in risk stratification for AF recurrence after RFCA.

Previous studies have reported various biomarkers of AF, such as hs-CRP (19), MMP-9 (18), N-terminal pro-B type natriuretic peptide (NT-proBNP) (20), bone morphogenic protein-10 (BMP10) (14), growth differentiation factor (GDF-15) (21), and serum soluble ST2 (sST2) (22). The appearance of AF is closely related to atrial structural remodeling and electrical remodeling. In the meantime, some studies also suggested that inflammation may play an important role in atrial remodeling (23–25). hs-CRP is a biological marker of local and systemic inflammation (19, 26). Many studies have shown that elevated levels of hs-CRP are independently associated with an increased risk of incident AF and AF recurrence after catheter ablation (26). Therefore, hs-CRP was used as a positive reference for predicting AF recurrence in our study compared with TIMP1. Our results showed that baseline TIMP-1 levels were associated with AF recurrence after a median follow-up period of 30 months. This TIMP-1 activity could help us understand AF pathogenesis and help predict cardiovascular risk and event recurrence. At the same time, high TIMP-1 levels and combined with women, age < 65 years, HTN, BMI ≥ 24 kg/m^2^, low CHA2DS2-VASc score, low HAS-BLED score, and high EHRA score will have a tendency to AF recurrence after RFCA.

## Conclusion

In a monocentric cohort of AF patients, baseline serum TIMP-1 levels before the initial RFCA procedure had an independent prognostic value in predicting long-term recurrence. Patients with a high TIMP-1 level were related to a higher risk of recurrent AF.

## Limitations

There are four limitations to our study. First, we measured serum TIMP-1 levels only at baseline and, thus, could not assess the effects of postoperative TIMP-1 levels, or the effect of the changes in TIMP-1 levels over time. Second, 24-h Holter monitoring may lead to an underestimation of the recurrence rates compared with the implanted loop recorder. Third, TIMP1 is not a routine laboratory examination in hospitals. Fourth, the study sample included patients with AF after RFCA and is a single-center study with a small sample size, and the generalizability of the results to other populations is unclear.

## Data availability statement

The raw data supporting the conclusions of this article will be made available by the authors, without undue reservation.

## Ethics statement

The studies involving human participants were reviewed and approved by the Beijing Anzhen Hospital Ethics Committee (Approval No: 2022042X). The patients/participants provided their written informed consent to participate in this study.

## Author contributions

HL and XZ designed the studies and wrote the manuscript. HL performed ELISA experiments. WS and ZeW contributed to the data collection. ZiW, XD, and JC contributed to the follow-up. YuW contributed to the ultrasound scan. XL, XW, and JG contributed to the data analysis. YoW designed and revised the manuscript. All authors contributed to the manuscript revision and approved the submitted version.

## Abbreviations

TIMP-1: tissue inhibitor of metalloproteinase-1
MMP-9: matrix metalloproteinase-9
AF: atrial fibrillation
RFCA: radiofrequency catheter ablation
BMI: body mass index
hs-CRP: high-sensitivity C-reactive protein
CPVI: circumferential pulmonary vein isolation
LA: left atrium
ECG: electrocardiogram
ROC: receiver operating characteristic
ECM: extracellular matrix.

## References

[1] Baman JR Passman RS. Atrial fibrillation. *JAMA.* (2021) 325:2218. 10.1001/jama.2020.23700 34061143

[2] Li D Nie J Han Y Ni L. Epigenetic mechanism and therapeutic implications of atrial fibrillation. *Front Cardiovasc Med.* (2021) 8:763824. 10.3389/fcvm.2021.763824 35127848 PMC8815458

[3] Mujovic N Marinkovic M Lenarczyk R Tilz R Potpara TS. Catheter ablation of atrial fibrillation: an overview for clinicians. *Adv Ther.* (2017) 34:1897–917. 10.1007/s12325-017-0590-z 28733782 PMC5565661

[4] Ai Y Xing Y Yan L Ma D Gao A Xu Q Atrial fibrillation and depression: a bibliometric analysis from 2001 to 2021. *Front Cardiovasc Med.* (2022) 9:775329. 10.3389/fcvm.2022.775329 35252380 PMC8888833

[5] Kuck KH Lebedev DS Mikhaylov EN Romanov A Geller L Kalejs O Catheter ablation or medical therapy to delay progression of atrial fibrillation: the randomized controlled atrial fibrillation progression trial (ATTEST). *Europace.* (2021) 23:362–9. 10.1093/europace/euaa298 33330909 PMC7947582

[6] Lau DH Linz D Sanders P. New findings in atrial fibrillation mechanisms. *Card Electrophysiol Clin.* (2019) 11:563–71. 10.1016/j.ccep.2019.08.007 31706465

[7] Fragao-Marques M Miranda I Martins D Barroso I Mendes C Pereira-Neves A Atrial matrix remodeling in atrial fibrillation patients with aortic stenosis. *BMC Cardiovasc Disord.* (2020) 20:468. 10.1186/s12872-020-01754-0 33129260 PMC7603735

[8] Sygitowicz G Maciejak-Jastrzebska A Sitkiewicz D. A review of the molecular mechanisms underlying cardiac fibrosis and atrial fibrillation. *J Clin Med.* (2021) 10:4430. 10.3390/jcm10194430 34640448 PMC8509789

[9] Nattel S. Molecular and cellular mechanisms of atrial fibrosis in atrial fibrillation. *JACC Clin Electrophysiol.* (2017) 3:425–35. 10.1016/j.jacep.2017.03.002 29759598

[10] Rafaqat S Sharif S Majeed M Naz S Manzoor F Rafaqat S. Biomarkers of metabolic syndrome: role in pathogenesis and pathophysiology of atrial fibrillation. *J Atr Fibrillation.* (2021) 14:20200495. 10.4022/jafib.20200495 34950373 PMC8691267

[11] Tsiachris D Giannopoulos G Deftereos S Kossyvakis C Tsioufis C Siasos G Biomarkers determining prognosis of atrial fibrillation ablation. *Curr Med Chem.* (2019) 26:925–37. 10.2174/0929867325666180320122930 29557741

[12] Marin F Roldan V Climent V Garcia A Marco P Lip GY. Is thrombogenesis in atrial fibrillation related to matrix metalloproteinase-1 and its inhibitor, TIMP-1? *Stroke.* (2003) 34:1181–6. 10.1161/01.STR.0000065431.76788.D9 12663879

[13] Kalman JM Kumar S Sanders P. Markers of collagen synthesis, atrial fibrosis, and the mechanisms underlying atrial fibrillation. *J Am Coll Cardiol.* (2012) 60:1807–8. 10.1016/j.jacc.2012.06.049 23040578

[14] Reyat JS Chua W Cardoso VR Witten A Kastner PM Kabir SN Reduced left atrial cardiomyocyte PITX2 and elevated circulating BMP10 predict atrial fibrillation after ablation. *JCI Insight.* (2020) 5:e139179. 10.1172/jci.insight.139179 32814717 PMC7455124

[15] Kim SK Park JH Kim JY Choi JI Joung B Lee MH High plasma concentrations of transforming growth factor-beta and tissue inhibitor of metalloproteinase-1: potential non-invasive predictors for electroanatomical remodeling of atrium in patients with non-valvular atrial fibrillation. *Circ J.* (2011) 75:557–64. 10.1253/circj.CJ-10-0758 21186331

[16] Choi JI Baek YS Roh SY Piccini JP Kim YH. Chromosome 4q25 variants and biomarkers of myocardial fibrosis in patients with atrial fibrillation. *J Cardiovasc Electrophysiol.* (2019) 30:1904–13. 10.1111/jce.14104 31393025

[17] Li CY Zhang JR Hu WN Li SN. Atrial fibrosis underlying atrial fibrillation (Review). *Int J Mol Med.* (2021) 47:9. 10.3892/ijmm.2020.4842 33448312 PMC7834953

[18] Wu G Wang S Cheng M Peng B Lian J Huang H The serum matrix metalloproteinase-9 level is an independent predictor of recurrence after ablation of persistent atrial fibrillation. *Clinics.* (2016) 71:251–6. 10.6061/clinics/2016(05)02 27276393 PMC4874263

[19] Meyre PB Sticherling C Spies F Aeschbacher S Blum S Voellmin G C-reactive protein for prediction of atrial fibrillation recurrence after catheter ablation. *BMC Cardiovasc Disord.* (2020) 20:427. 10.1186/s12872-020-01711-x 32993521 PMC7526257

[20] Staszewsky L Meessen J Novelli D Wienheus-Thelen UH Disertori M Maggioni AP Total NT-proBNP, a novel biomarker related to recurrent atrial fibrillation. *BMC Cardiovasc Disord.* (2021) 21:553. 10.1186/s12872-021-02358-y 34798808 PMC8603582

[21] Wei Y Liu S Yu H Zhang Y Gao W Cui M The predictive value of growth differentiation factor-15 in recurrence of atrial fibrillation after catheter ablation. *Mediators Inflamm.* (2020) 2020:8360936. 10.1155/2020/8360936 32904560 PMC7456492

[22] AlTurki A. Soluble ST2 in paroxysmal atrial fibrillation: a new biomarker that predicts recurrence? *Korean Circ J.* (2018) 48:930–2. 10.4070/kcj.2018.0183 30238710 PMC6158446

[23] Hu YF Chen YJ Lin YJ Chen SA. Inflammation and the pathogenesis of atrial fibrillation. *Nat Rev Cardiol.* (2015) 12:230–43. 10.1038/nrcardio.2015.2 25622848

[24] Richter B Gwechenberger M Socas A Zorn G Albinni S Marx M Time course of markers of tissue repair after ablation of atrial fibrillation and their relation to left atrial structural changes and clinical ablation outcome. *Int J Cardiol.* (2011) 152:231–6. 10.1016/j.ijcard.2010.07.021 20692054

[25] Baz L Grun K Diab M Pfeil A Jung C Mobius-Winkler S Prediction of one- and two-year mortality after transcatheter aortic valve implantation: proposal of a fast sum-score system integrating a novel biomarker of cardiac extracellular matrix accumulation and fibrosis. *Rev Cardiovasc Med.* (2022) 23:62. 10.31083/j.rcm2302062 35229553

[26] Liu T Li G Li L Korantzopoulos P. Association between C-reactive protein and recurrence of atrial fibrillation after successful electrical cardioversion: a meta-analysis. *J Am Coll Cardiol.* (2007) 49:1642–8. 10.1016/j.jacc.2006.12.042 17433956

