# Supplementary material for: Higher serum tissue inhibitor of metalloproteinase-1 predicts atrial fibrillation recurrence after radiofrequency catheter ablation
Source: Front Cardiovasc Med. 2022 Oct 13;9:961914. doi: 10.3389/fcvm.2022.961914 (PMC9606231; doi:10.3389/fcvm.2022.961914)

**supplemental table 1**

**Primary screening of risk factors for AF recurrence by univariate Cox analysis**

| **Characteristics** | **AUC** | **ROC best cut-off value** | **Univariate Cox** |
| --- | --- | --- | --- |
| **WBC, 10^9^/l** | 0.476 | 5.74 | 0.118 |
| **RBC, 10^9^/l** | 0.499 | 5.49 | 0.09 |
| **Hb, g/l** | 0.472 | 172.5 | 0.193 |
| **PLT, 10^9^/l** | 0.59 | 196.5 | 0.023 |
| **CREA, umol/l** | 0.372 | 57.25 | 0.625 |
| **Glu, mmol/l** | 0.481 | 5.58 | 0.373 |
| **GA, %** | 0.458 | 12.09 | 0.31 |
| **hsCRP, ng/ml** | 0.574 | 1.12 | 0.011 |
| **TIMP-1, ng/ml** | 0.573 | 124.15 | 0.009 |
| **BNP, pg/ml** | 0.511 | 113.5 | 0.199 |
| **LVSDD, mm** | 0.465 | 33.5 | 0.115 |
| **LVEDD, mm** | 0.431 | 56.5 | 0.486 |
| **TBIL, umol/l** | 0.511 | 18.19 | 0.132 |
| **ALT, U/l** | 0.530 | 26.5 | 0.419 |
| **AST, U/l** | 0.503 | 27.5 | 0.225 |
| **GGT, U/l** | 0.501 | 17.5 | 0.292 |
| **TP, g/l** | 0.472 | 62.9 | 0.258 |
| **Alb, g/l** | 0.460 | 47.75 | 0.364 |
| **Glo, g/l** | 0.502 | 22.55 | 0.193 |
| **TCHO, mmol/l** | 0.514 | 5.94 | 0.04 |
| **LDL-c, mmol/l** | 0.514 | 2.44 | 0.472 |
| **Ddimer, ng/ml** | 0.501 | 108 | 0.565 |
| **Age, years** | 0.496 | 65 | 0.767 |
| **HCY, umol/l** | 0.396 | 15 | 0.218 |
| **LAd, mm** | 0.459 | 40 | 0.618 |
| **LVEF, %** | 0.524 | 50 | 0.828 |
| **BMI, kg/m2** | 0.505 | 24 | 0.946 |
| **TG, mmol/l** | 0.428 | 1.7 | 0.344 |
| **Substrate modification** |  |  | 0.553 |
| **EHRA score** |  |  | 0.828 |
| **HASBLED score** |  | 3 | 0.38 |
| **CHA2DS2VASc score** |  | 2 | 0.96 |
| **βblocker** |  |  | 0.777 |
| **CCB** |  |  | 0.909 |
| **ACEI** |  |  | 0.231 |
| **Statins** |  |  | 0.432 |
| **Diuretic** |  |  | 0.433 |
| **Drinking** |  |  | 0.924 |
| **Smoking** |  |  | 0.113 |
| **Stroke** |  |  | 0.566 |
| **DM** |  |  | 0.563 |
| **HTN** |  |  | 0.842 |
| **CAD** |  |  | 0.761 |
| **Gender** |  |  | 0.121 |

WBC: white blood cell; RBC: red blood cell; PLT: platelet count; Hb: hemoglobin; CREA: creatinine; Glu: fasting blood glucose; GA: glycated albumin; HCY: homocysteine; ALT: alanine aminotransferase; AST: aspartate transaminase; GGT: gamma-glutamyl transpeptidase; TP: total protein; Alb: albumin; Glo: globulin; Tbil: total bilirubin; TG: triacylglycerol; Tcho: total cholesterol; LDL-c: low-density lipoprotein cholesterol; TIMP-1: tissue inhibitors of metalloproteinase-1; hsCRP: high-sensitivity C-reactive protein; BNP: B-type natriuretic peptide; BMI: body mass index; CAD: coronary artery disease; HTN: hypertension; DM: diabetes mellitus; ACEI: angiotensin-converting enzyme inhibitors; ARB: angiotensin receptor blocker; CCB: calcium channel blocker; LAd: Left atrium diameter; LVEF: left ventricular ejection fraction; LVEDD: left ventricular end-diastolic dimension; LVESD: left ventricular end-systolic dimension.

**Supplementary figure.1**

**Kaplan-Meier survival curves for freedom from AF recurrence stratified by substrate modification.**


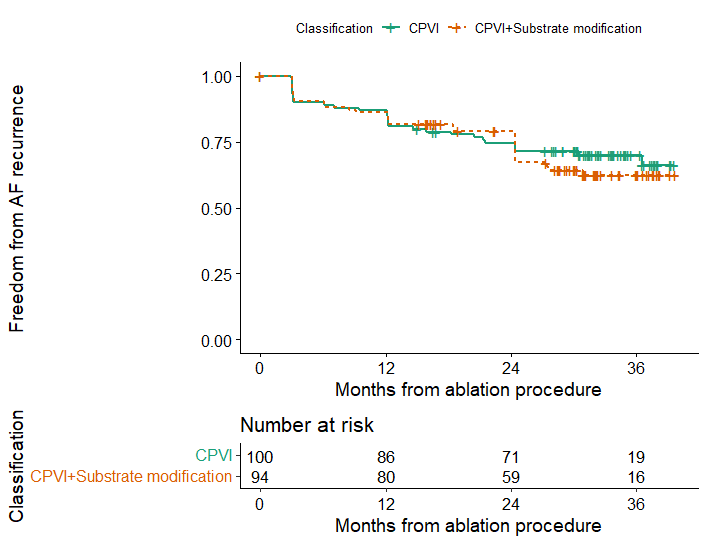

Supplement: Supplementary file 1 [file Data_Sheet_1.docx]
