# Supplementary material for: Higher serum tissue inhibitor of metalloproteinase-1 predicts atrial fibrillation recurrence after radiofrequency catheter ablation
Source: Front Cardiovasc Med. 2022 Oct 13;9:961914. doi: 10.3389/fcvm.2022.961914 (PMC9606231; doi:10.3389/fcvm.2022.961914)
Supplement: Supplementary file 2 [file Table_1.pdf]

Supplemental Table 1

Primary screening of risk factors for AF recurrence by univariate Cox analysis

Characteristics	AUC	ROC best cut-off value	Univariate Cox
**WBC, 10^9/l**	0.476	5.74	0.118
**RBC, 10^9/l**	0.499	5.49	0.09
**Hb, g/l**	0.472	172.5	0.193
**PLT, 10^9/l**	0.59	196.5	0.023
**CREA, umol/l**	0.372	57.25	0.625
**Glu, mmol/l**	0.481	5.58	0.373
**GA, %**	0.458	12.09	0.31
**hsCRP, ng/ml**	0.574	1.12	0.011
**TIMP-1, ng/ml**	0.573	124.15	0.009
**BNP, pg/ml**	0.511	113.5	0.199
**LVSDD, mm**	0.465	33.5	0.115
**LVEDD, mm**	0.431	56.5	0.486
**TBIL, umol/l**	0.511	18.19	0.132
**ALT, U/l**	0.530	26.5	0.419
**AST, U/l**	0.503	27.5	0.225
**GGT, U/l**	0.501	17.5	0.292
**TP, g/l**	0.472	62.9	0.258
**Alb, g/l**	0.460	47.75	0.364
**Glo, g/l**	0.502	22.55	0.193
**TCHO, mmol/l**	0.514	5.94	0.04
**LDL-c, mmol/l**	0.514	2.44	0.472
**Ddimer, ng/ml**	0.501	108	0.565
**Age, years**	0.496	65	0.767
**HCY, umol/l**	0.396	15	0.218
**LAd, mm**	0.459	40	0.618
**LVEF, %**	0.524	50	0.828
**BMI, kg/m2**	0.505	24	0.946
**TG, mmol/l**	0.428	1.7	0.344
**Substrate modification**			0.553
**EHRA score**			0.828
**HASBLED score**		3	0.38
**CHA2DS2VASc score**		2	0.96
**βblocker**			0.777
**CCB**			0.909
**ACEI**			0.231
**Statins**			0.432
**Diuretic**			0.433
**Drinking**			0.924

**Smoking**	0.113
**Stroke**	0.566
**DM**	0.563
**HTN**	0.842
**CAD**	0.761
**Gender**	0.121

WBC: white blood cell; RBC: red blood cell; PLT: platelet count; Hb: hemoglobin; CREA: creatinine; Glu: fasting blood glucose; GA: glycated albumin; HCY: homocysteine; ALT: alanine aminotransferase; AST: aspartate transaminase; GGT: gamma-glutamyl transpeptidase; TP: total protein; Alb: albumin; Glo: globulin; Tbil: total bilirubin; TG: triacylglycerol; Tcho: total cholesterol; LDL-c: low-density lipoprotein cholesterol; TIMP-1: tissue inhibitors of metalloproteinase-1; hsCRP: high-sensitivity C-reactive protein; BNP: B-type natriuretic peptide; BMI: body mass index; CAD: coronary artery disease; HTN: hypertension; DM: diabetes mellitus; ACEI: angiotensin-converting enzyme inhibitors; ARB: angiotensin receptor blocker; CCB: calcium channel blocker; LAd: Left atrium diameter; LVEF: left ventricular ejection fraction; LVEDD: left ventricular end-diastolic dimension; LVESD: left ventricular end-systolic dimension.

Supplementary Figure.1

Kaplan-Meier survival curves for freedom from AF recurrence stratified by substrate modification.
